# Supplementary material for: Reactivation of Varicella-Zoster Virus in Patients with Lung Cancer Receiving Immune Checkpoint Inhibitors: Retrospective Nationwide Population-Based Cohort Study from South Korea
Source: Cancers (Basel). 2024 Apr 14;16(8):1499. doi: 10.3390/cancers16081499 (PMC11048333; doi:10.3390/cancers16081499)
Supplement: Supplementary file 1 [file cancers-16-01499-s001.zip › cancers-2877934-supplementary.pdf]

Supplemental Table S1. 1:1 Propensity score matching between ICIs and non-ICIs groups using the age, sex, comorbidities, and concomitant use of immunosuppressive drugs.

	Before matching			After matching		
	**ICIs (n = 897)**	**Non-ICIs (n = 50,124)**	***p*-value**	**ICIs (n = 652)**	**Non-ICIs (n = 652)**	***p*-value**
**Age (year), n (%)**			0.008			0.262
< 40 (%)	6 (0.7)	475 (0.9)		6 (0.9)	10 (1.5)	
40–50 (%)	44 (4.9)	2,019 (4.0)		40 (6.1)	34 (5.2)	
50–60 (%)	156 (17.4)	8,040 (16.0)		132 (20.2)	122 (18.7)	
60–70 (%)	303 (33.8)	18,395 (36.7)		218 (33.4)	226 (34.6)	
70–80 (%)	298 (33.2)	17,555 (35.0)		220 (33.7)	241 (36.9)	
≥ 80 (%)	90 (10.0)	3,640 (7.3)		36 (5.52)	19 (2.9)	
**Sex, n (%)**			0.3112			0.375
Male	678 (75.6)	37,109 (74.0)		489 (75.1)	482 (73.9)	
Female	219 (24.4)	13,015 (26.0)		163 (24.9)	170 (26.1)	
**Comorbidity, n (%)**						
Diabetes	350 (39.0)	21,708 (43.3)	0.011	240 (36.8)	247 (37.8)	0.461
Cardiovascular disease*	301 (33.6)	20,791 (41.5)	<0.001	226 (34.6)	234 (35.8)	0.125
Chronic lung diseases	601 (67.0)	36,234 (72.3)	0.001	423 (64.8)	434 (66.5)	0.798
Chronic kidney diseases	59 (6.6)	2,751 (5.5)	0.179	38 (5.8)	39 (6.6)	0.174
Chronic liver diseases	25 (2.8)	1,404 (2.8)	1.000	13 (1.9)	16 (2.4)	0.366
Rheumatic diseases	48 (5.4)	3,221(6.4)	0.217	32 (4.9)	33 (5.0)	1.000
**Concomitant use of immunosuppressive drugs, n (%)**						
Immunosuppressant	31 (3.5)	2,428 (4.8)	0.065	18 (2.7)	21 (3.3)	0.210
Steroid**	34 (3.8)	3,386 (6.8)	0.001	23 (3.5)	24 (3.7)	1.000
